# SGCLDGA: unveiling drug–gene associations through simple graph contrastive learning

**DOI:** 10.1093/bib/bbae231

**Published:** 2024-05-16

**Authors:** Yanhao Fan, Che Zhang, Xiaowen Hu, Zhijian Huang, Jiameng Xue, Lei Deng

**Affiliations:** School of Computer Science and Engineering, Central South University, 410075, Changsha, China; School of software, Xinjiang University, 830046, Urumqi, China; School of Computer Science and Engineering, Central South University, 410075, Changsha, China; School of Computer Science and Engineering, Central South University, 410075, Changsha, China; School of Computer Science and Engineering, Central South University, 410075, Changsha, China; School of Computer Science and Engineering, Central South University, 410075, Changsha, China

**Keywords:** drug–gene association, singular value decomposition, contrastive learning, graph neural network

## Abstract

Drug repurposing offers a viable strategy for discovering new drugs and therapeutic targets through the analysis of drug–gene interactions. However, traditional experimental methods are plagued by their costliness and inefficiency. Despite graph convolutional network (GCN)-based models’ state-of-the-art performance in prediction, their reliance on supervised learning makes them vulnerable to data sparsity, a common challenge in drug discovery, further complicating model development. In this study, we propose SGCLDGA, a novel computational model leveraging graph neural networks and contrastive learning to predict unknown drug–gene associations. SGCLDGA employs GCNs to extract vector representations of drugs and genes from the original bipartite graph. Subsequently, singular value decomposition (SVD) is employed to enhance the graph and generate multiple views. The model performs contrastive learning across these views, optimizing vector representations through a contrastive loss function to better distinguish positive and negative samples. The final step involves utilizing inner product calculations to determine association scores between drugs and genes. Experimental results on the DGIdb4.0 dataset demonstrate SGCLDGA’s superior performance compared with six state-of-the-art methods. Ablation studies and case analyses validate the significance of contrastive learning and SVD, highlighting SGCLDGA’s potential in discovering new drug–gene associations. The code and dataset for SGCLDGA are freely available at https://github.com/one-melon/SGCLDGA.

## INTRODUCTION

Traditional drug development and discovery has been a time-consuming, high-investment, high-risk endeavor [1]. It is usually accompanied by a failure rate of upwards of 96% [2], and the cost of bringing a new drug to market is estimated to be as high as $1.3 billion [3]. Drug repurposing is one way to address this challenge. By revealing drug–gene interactions, we will not only be able to uncover new drugs, but also identify potential therapeutic targets [4–6]. Research findings indicate that drugs designed to treat the same disease often exhibit similar chemical structures, while cell lines with comparable gene expression profiles demonstrate analogous chemical responses to the same drug [7–9]. This observation underscores the potential for uncovering new drug–gene associations and identifying novel drug targets through the analysis of modular relationships between disease gene expression profiles and drugs. Such insights hold promise for repurposing existing drugs for the treatment of other diseases. For instance, crizotinib exerts kinase-dependent cytotoxicity through dual inhibition of microtubule protein polymerization and topoisomerase II, making it suitable not only for the treatment of non-small cell lung cancer (NSCLC), but also for the treatment of multiple myeloma [10].

Nonetheless, conventional biological experiments are not only time-consuming and costly but also inefficient and susceptible to external environmental factors [11, 12]. With technological advancements, the field of bioinformatics has amassed a wealth of multi-source data, providing an opportunity for the development of efficient and cost-effective computational methods [13–16]. Machine learning is a technique that transforms input data into output results, which mainly consists of a training step and an inference step [17]. Liu *et al*. [18] developed SMALF, a computational framework for predicting micro RNAs (miRNAs) related to diseases, which fused latent features with original features from the miRNA–disease association matrix, employing XGBoost to deduce unknown miRNA–disease associations. To identify disease-related long noncoding RNAs (lncRNAs), Wu *et al*. [19] proposed GAERF, a classification method that utilized machine learning techniques based on graph autoencoder (GAE) and random forest. Sun *et al*. [20] introduced RWRlncD, a global network-based computational framework integrating lncRNA-disease networks, disease similarity networks and lncRNA functional similarity networks to rank candidate lncRNAs for specific diseases. Li *et al*. [21] constructed a label propagation model (LPLNS) based on the linear neighborhood similarity of lncRNA and disease. Traditional machine learning methods have limitations in processing raw data. In contrast, deep learning, through the technique of representation learning with hierarchical features, can transform raw inputs into more advanced and abstract representations [22]. Palhamkhani *et al*. [23] proposed a deep learning-based DeepCompoundNet model that integrates protein features, drug properties and various interaction data to predict chemical–protein interactions. Xuan *et al*. [24] introduced CNNMDA, a prediction method for disease-related miRNAs based on network representation learning and convolutional neural networks.

Graph convolutional networks (GCNs) have proven powerful in learning graph data with irregularities, aggregating neighbor node information to understand the global structure of the graph [25]. Li *et al*. [26] proposed HGCNMDA, a method based on graph neural networks, utilizing node2vec and GCN to learn joint features of miRNAs and diseases from a protein–protein Interaction network. Yu *et al*. [27] introduced LAGCN, a method that learns and integrates drug and disease embeddings using graph convolution and attention from a heterogeneous network. Li *et al*. [28] proposed NIMCGCN, a method leveraging GCNs to learn hidden features of miRNA and disease from their similarity networks. While GCN-based models showcase state-of-the-art performance in association with prediction problems, most follow a supervised learning paradigm, making them susceptible to data sparsity [29]. Data sparsity can compromise the generalization ability of these models, impacting prediction accuracy and reliability, particularly in the drug discovery field.

In recent years, contrastive learning, a self-supervised learning method, has demonstrated significant success in modeling graph structures and found widespread applications in various graph data domains. Particularly, it has proven effective in association prediction tasks by establishing a clear separation between positive and negative samples, even in scenarios with sparse supervision signals. Liu *et al*. [30] introduced MPCLCDA, a model incorporating automatic meta-path selection and contrastive learning to predict potential circular RNA (circRNA)–disease associations. Ai *et al*. [31] proposed GDCL-NcDA, leveraging deep GCNs and multiple attention mechanisms to reconstruct multi-source heterogeneous networks. GDCL-NcDA predicts potential noncoding RNA–disease associations using deep matrix factorization (MF) and contrastive learning. Zhang *et al*. [32] presented NCH-DDA, a DDA model based on neighborhood contrastive learning. It extracts neighborhood features of drugs and diseases from various spaces, including heterogeneous networks and similarity networks. Dehghan *et al*. [33] explore how contrast loss functions can be utilized in conjunction with task prediction loss functions to help learn more robust models. Through contrastive learning, it fuses these features to obtain universal drug and disease features, enabling the prediction of new drug–disease associations. However, it is worth noting that random perturbation in graph augmentation may inadvertently erase valuable structural information, leading to biased representation learning. Additionally, representation contrast schemes guided by heuristics heavily rely on the design of the view generator, potentially limiting the model’s generalization ability.

In this paper, we propose SGCLDGA, a computational model leveraging graph neural networks and self-supervised contrastive learning for the prediction of unknown drug–gene associations. SGCLDGA employs GCNs to acquire vector representations of drugs and genes, using inner product calculation for association scores. To enhance the graph and generate multi-view representations, SGCLDGA utilizes singular value decomposition (SVD) and performs contrastive learning across different views, enhancing the quality of vector representations.

Our main contributions are listed below:

- We have proposed a lightweight yet powerful graph comparison learning framework applied to the prediction of drug–gene associations, to address key challenges associated with this task.
- We propose an effective and efficient model, SGCLDGA, guided by SVD for graph enhancement. This approach effectively preserves important information regarding gene–drug associations and eliminates the need to generate two manually enhanced views, addressing the shortcomings of randomly perturbed graph enhancement and heuristic-based contrast view generators.
- Compared with existing GCL-based methods, our approach improves training efficiency, and notably outperforms similarity-based methods in terms of training efficiency, particularly with larger datasets.
- A series of experiments have demonstrated the performance advantages of our SGCLDGA model. In-depth analysis confirms the rationality and robustness of SGCLDGA, and underscores its practical value.

## METHODS

### Overview of SGCLDGA

**Figure 1:**
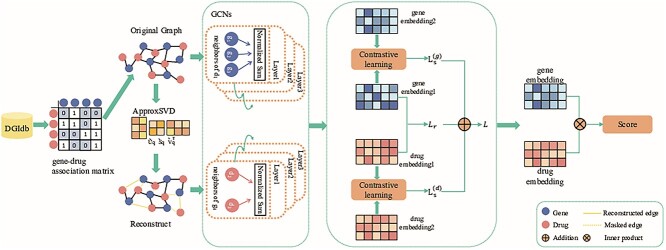
Architecture overview of SGCLDGA. It contains four distinct modules for effective drug–gene association prediction: (1) Local Neighbor Encoding Module: employs a GCN to derive vector representations of drugs and genes from the original drug–gene bipartite graph, capturing local relationships. (2) Collaborative Relation Learning Module: utilizes SVD to enrich the graph and generate multiple views, enhancing collaborative relations. (3) Contrastive Learning Module: SGCLDGA conducts contrastive learning across diverse views, optimizing vector representations through a contrastive loss function to distinguish positive and negative samples. (4) Prediction Module: integrates drug and gene embeddings from prior modules, employing inner product calculations to determine association scores between drugs and genes, culminating in the final predictions. This multi-faceted approach ensures robust and accurate predictions of drug–gene associations.

The overall architecture of SCGCLDGA is illustrated in Figure 1. Initially, SCGCLDGA employs GCNs to acquire embedding representations for both genes and drugs. To create multi-view representations of gene and drug nodes, the model applies SVD to enrich the graph, resulting in distinct subgraphs, each representing a unique view. Subsequently, SCGCLDGA engages in contrastive learning across these different views, utilizing a contrastive loss function to optimize the vector representations. This process enhances the quality and robustness of the representations. Finally, SCGCLDGA computes the association between drugs and genes using inner product calculations.

### Dataset

The drug–gene Interaction Database 4.0 (DGIdb4.0) [34] stands as a user-friendly search tool and extensive database, furnishing information on associations between genes and drugs, whether established or potential. As of now, DGIdb 4.0 encompasses data on 10 606 druggable genes and 54 591 drug–gene interactions, spanning 41 102 genes and 14 449 drugs. For our experimental data, we specifically curated 46 892 established drug–gene associations from DGIdb 4.0, involving 10 690 drugs and 3227 genes.

### Local neighborhood encoding module

We denoted the drug set as $\mathbf{D}=\{d_{1},d_{2},\ldots ,d_{m}\}$ and the gene set as $\mathbf{G}=\{g_{1},g_{2},\ldots ,g_{n}\}$. Here, $m$ is the number of drugs, and $n$ is the number of genes in the dataset. We constructed the association matrix $\mathbf{A} \in \mathbf{R}^{m\times n}$, where $\mathbf{A}(d_{i},g_{j})$ indicated the association strength between drug $d_{i}$ and gene $g_{j}$. We assigned $\mathbf{A}(d_{i},g_{j})=1$ if drug $d_{i}$ and gene $g_{j}$ had a known association, and $\mathbf{A}(d_{i},g_{j})=0$ otherwise.

We assigned an embedding vector $e_{i}^{(d)}, e_{j}^{(g)}\in \mathbf{R}^{d}$ to each drug $d_{i}$ and each gene $g_{j}$, where d represents the size of the embedding. These vectors form the drug embedding matrix $\mathbf{E}^{(d)} \in \mathbf{R}^{m \times d}$ and the gene embedding matrix $\mathbf{E}^{(g)} \in \mathbf{R}^{n \times d}$, where $m$ and $n$ are the number of drugs and genes, respectively. Then, inspired by the method of hypergraph contrastive collaborative filtering [35], we use a two-layer GCN to aggregate the neighbor information of each node in the graph. Specifically, the aggregation function at the l-th layer can be expressed as 


(1) \begin{align*}& \left\{ \begin{aligned} z_{i,l}^{(d)}=\sigma(p(\tilde{\mathbf{A}}_{i,:})\cdot\mathbf{E}_{l-1}^{(g)})\\ z_{j,l}^{(g)}=\sigma(p(\tilde{\mathbf{A}}_{:,j})\cdot\mathbf{E}_{l-1}^{(d)})\\ \end{aligned}\right.,\end{align*}

where $z_{i,l}^{(d)}$ and $z_{j,l}^{(g)}$ represent the l-th layer aggregated embedding vectors of drug $d_{i}$ and gene $g_{j}$. $\sigma{(\cdot )}$ is the nonlinear activation function, $\tilde{\mathbf{A}}$ signifies the normalized adjacency matrix and $p{(\cdot )}$ indicates the dropout, which is employed to prevent model overfitting. The final embeddings $e_{i}^{(d)}$ and $e_{j}^{(g)}$ of drug $d_{i}$ and gene $g_{j}$ are obtained by summing the embeddings of all layers: 


(2) \begin{align*}& \left\{ \begin{aligned} e_{i}^{(d)}=\sum\limits_{l=0}^{L}z_{i,l}^{(d)}\\ e_{j}^{(g)}=\sum\limits_{l=0}^{L}z_{i,l}^{(g)}\\ \end{aligned}\right.\end{align*}

### Collaborative relation learning

We use the SVD scheme [36, 37] to enhance our SGCLDGA model, which enables it to leverage the global structure information in graph contrastive learning. The SVD scheme extracts collaborative signals from the global perspective by performing SVD on the normalized adjacency matrix $\tilde{\mathbf{A}}$. Specifically, we decompose $\tilde{\mathbf{A}}$ as $\tilde{\mathbf{A}}=\mathbf{USV}^{T}$,where $\mathbf{U/V}$ are $m\times m/n\times n$ orthonormal matrices, whose columns are the eigenvectors of $\tilde{\mathbf{A}}$’s row–row and column–column correlation matrices, respectively. $\mathbf{S}$ is an $m \times n$ diagonal matrix, whose diagonal elements are the singular values of $\tilde{\mathbf{A}}$. To preserve the principal components of the matrix, we only select the largest $t$ singular values and their corresponding eigenvectors, and then approximate $\tilde{\mathbf{A}}$ with the truncated matrix $\grave{\mathbf{A}}=\mathbf{U}_{t}\mathbf{S}_{t}\mathbf{V}_{t}^{T}$, where $\mathbf{U}_{t} \in \mathbf{R}^{m \times t}$ and $\mathbf{V}_{t} \in \mathbf{R}^{n \times t}$ are the first $t$ columns of $\mathbf{U}$ and $\mathbf{V}$, respectively. $\mathbf{S}_{t} \in \mathbf{R}^{t \times t}$ is the diagonal matrix of the largest $t$ singular values.

However, performing the exact SVD on large matrices is very time-consuming. Therefore, we adopt a randomized SVD algorithm [38], which approximates the range of the input matrix with a low-rank orthonormal matrix, and then performs SVD on this smaller matrix.The approximated versions of $\mathbf{U}_{t}$, $\mathbf{S}_{t}$ and $\mathbf{V}_{t}$ are denoted by $\grave{\mathbf{U}_{t}}$, $\grave{\mathbf{S}_{t}}$ and $\grave{\mathbf{V}_{t}}$, respectively. The desired rank for the decomposed matrices is indicated by $t$: 


(3) \begin{align*}& \grave{\mathbf{U}}_{t},\grave{\mathbf{S}}_{t},\grave{\mathbf{V}}_{t}^{T}=ApproxSVD(\tilde{\mathbf{A}},t), \grave{\mathbf{A}}^{(SVD)}=\grave{\mathbf{U}}_{t}\grave{\mathbf{S}}_{t}\grave{\mathbf{V}}_{t}^{T}\end{align*}

After getting the approximated adjacency matrix $\grave{\mathbf{A}}^{(SVD)}$, we conduct message propagation on the reconstructed drug–gene relation graph in each layer: 


(4) \begin{align*}& \left\{ \begin{aligned} \mathbf{h}_{i,l}^{(d)} = \sigma{(\grave{\mathbf{A}}^{(SVD)}_{i,:} \cdot \mathbf{E}_{l-1}^{(g)})} \\ \mathbf{h}_{j,l}^{(g)} = \sigma{(\grave{\mathbf{A}}^{(SVD)}_{:,j} \cdot \mathbf{E}_{l-1}^{(d)})} \\ \end{aligned} \right.,\end{align*}

where $\mathbf{h}_{i,l}^{(d)}$ and $\mathbf{h}_{j,l}^{(g)}$ are the l-th layer aggregated embeddings of drugs and genes, which are generated by the newly constructed graph structure view. $\sigma{(\cdot )}$ is the nonlinear activation function.

### Contrastive learning module

In our proposed method, we adopt the SVD method to construct a new graph view, which can utilize the global collaborative relations of the graph to enhance the embedding representation ability of the main view. We treat the main view embeddings and the augmented view embeddings of the same drug or gene as positive sample pairs, and the main view embeddings and the augmented view embeddings of different drugs or genes as negative sample pairs. We use InfoNCE [39] to define our contrastive loss for drug and gene representations: 


(5) \begin{align*}& \left\{ \begin{aligned} \mathcal{L}_{s}^{(d)} = \sum_{i=0}^{I}\sum_{l=0}^{L}-log\frac{exp\Big(s(z_{i,l}^{(d)}, h_{i,l}^{(d)}/\tau)\Big)}{\sum_{i^{-}=0}^{I}exp\Big(s(z_{i,l}^{(d)}, h_{i^{-},l}^{(d)})/\tau\Big)} \\ \mathcal{L}_{s}^{(g)} = \sum_{j=0}^{J}\sum_{l=0}^{L}-log\frac{exp\Big(s(z_{j,l}^{(g)}, h_{j,l}^{(g)}/\tau)\Big)}{\sum_{j^{-}=0}^{J}exp\Big(s(z_{j,l}^{(g)}, h_{j^{-},l}^{(g)})/\tau\Big)} \\ \end{aligned} \right.,\end{align*}

where $s(\cdot )$ denotes the cosine similarity function and $\tau $ represents the tunable temperature hyperparameter to adjust the scale for softmax.To prevent overfitting, we make some nodes randomly inactive in each batch, so that they do not participate in contrastive learning.

### Prediction and optimization

To predict the association preference score between drugs and genes, we adopt an inner product-based method. Specifically, we first sum up all the embedding layers of drug $d_{i}$ and gene $g_{j}$, obtaining their final feature vectors $e_{i}^{(d)}$ and $e_{j}^{(g)}$. Then, we use the inner product of the feature vectors of drug $d_{i}$ and gene $g_{j}$ as their association preference score $P_{i,j}$: 


(6) \begin{align*}& P_{{i,j}}=e^{(d)T}_{i}e_{j}^{(g)}\end{align*}

Our model is trained by positive and negative sample pairs, where positive sample pairs are different views of the same drug and gene, and negative sample pairs are different views of different drugs and genes. we define our pair-wise loss as follows: 


(7) \begin{align*}& \mathcal{L}_{r} = \sum_{i=0}^{I}\sum_{s=1}^{S}max(0,1 - P_{r_{i,p_{s}}} + P_{r_{i,n_{s}}})\end{align*}

We jointly optimize the contrastive loss and the pair-wise loss: 


(8) \begin{align*}& \mathcal{L} = \mathcal{L}_{r} + \lambda_{1} \cdot (\mathcal{L}_{s}^{(d)} + \mathcal{L}_{s}^{(g)}) + \lambda_{2} \cdot ||\Theta||_{2}^{2},\end{align*}

where $\Theta $ denotes the weight decay regularization term. $\lambda _{1}$ and $\lambda _{2}$ are hyperparameters tuning the strength of self-supervised learning and L2 regularization, respectively.

## experiments and results

### Experiment setup

To assess the effectiveness of the SGCLDGA model, we employed 5-fold cross-validation (5-fold CV) on our experimental dataset. We randomly divided the 46 892 known gene–drug association pairs into five equal-sized subsets. In each iteration of the cross-validation, four subsets were used for training the model, while the remaining subset was used for testing.

To evaluate the performance of the SGCLDGA model, we utilized five commonly used metrics,including AUC (area under the receiver operating characteristics (ROC) curve), AUPR (Area Under the Precision–Recall Curve), recall (Recall), precision (Preci sion) and F1 (F1-score). The formulas for computing recall,precision and F1 are as follows: 


(9) \begin{align*} & TPR =\frac{TP}{TP +FN} \end{align*}

(10) \begin{align*} & FPR =\frac{FP}{TN +FP} \end{align*}

(11) \begin{align*} & Recall =\frac{TP}{TP+FN} \end{align*}

(12) \begin{align*} & Precision = \frac{TP}{TP+FP} \end{align*}

(13) \begin{align*} & \text{F1-score} =2\times \frac{Precision\times Recall}{Presion+Recall} \end{align*}

### Comparative experiment

In this section, we compare our method with the following methods.

- **LRGCPND** [40]: LRGCPND is the inaugural computational model for predicting ncRNA resistance, which employs a sequential approach to model the bipartite graph of ncRNA resistance. It begins by capturing the neighbor information representation through aggregation, followed by feature transformation via linear operations. Ultimately, the model utilizes residual links to make the final prediction, integrating information from earlier stages to enhance accuracy.
- **LightGCN** [41]: LightGCN is a variant of GCNs that simplifies the original architecture by focusing solely on the neighbor aggregation component.
- **LAGCN** [27]: LAGCN is a computational model designed for predicting new associations between drugs and diseases; the learning process involves multiple networks and employs a graph convolution algorithm to acquire embeddings of drugs and diseases. Subsequently, attention mechanisms are employed to integrate these embeddings, facilitating the prediction of novel associations.
- **MF** [42]: MF utilizes the similarities between drug and gene as underlying factors to forecast potential connections.
- **AGAEMD** [43]: AGAEMD is a computational model specifically designed for aggregating information in the miRNA–disease network. It achieves this by employing a node-level attention autoencoder, which enables the model to capture important features and patterns within the network. By leveraging this autoencoder, AGAEMD reconstructs the miRNA–disease associations network, providing a comprehensive understanding of the relationships between miRNAs and diseases.
- **MNGACDA** [44]: MNGACDA is a cutting-edge computational model that predicts the associations between circRNAs and drug sensitivity, utilizing multimodal networks, attention GAEs and inner product decoders to provide reliable predictions for biomedical screening.

**Figure 2:**
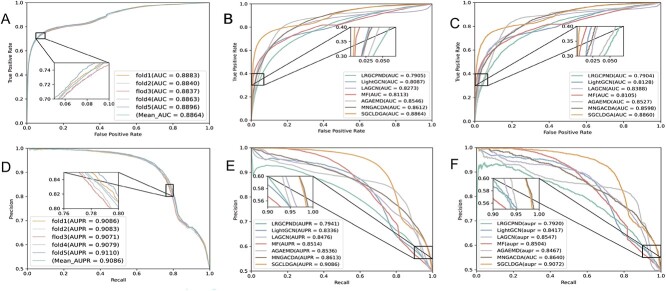
(**A**) ROC curves yielded by SGCLDGA in 5-fold CV. (**B**) ROC Curve of Comparative Experimental in 5-fold CV. (**C**) ROC Curve of Comparative Experimental on the validation set. (**D**) PR curves yielded by SGCLDGA in 5-fold CV. (**E**) PR Curve of Comparative Experimental in 5-fold CV. (**F**) PR Curve of Comparative Experimental on the validation set.

Table 1 presents the performance outcomes of SGCLDGA within the context of a 5-fold cross-validation. Additionally, Table 2 offers a comprehensive summary of our experimental analysis, illustrating the superior performance of our proposed SGCLDGA across five crucial evaluation metrics, namely, AUC, AUPR, Recall, Precision and F1-score. The graphical representations of the ROC and AUPR curves for SGCLDGA derived from the 5-fold cross-validation and comparative experiments are depicted in Figure 2 ADBE. The results underscore the outstanding stability and robustness of SGCLDGA throughout the 5-fold cross-validation process. Furthermore, SGCLDGA outperforms alternative methods in the comparative experiments, securing the most favorable outcomes. Notably, our approach, SGCLDGA, attains impressive performance metrics of 0.8863, 0.9076, 0.8279, 0.8368 and 0.8268 for AUC, AUPR, Recall, Precision and F1-score, respectively. These values exhibit a significant superiority over the corresponding metrics achieved by the sub-optimal method, surpassing them by margins of 2.9%, 5.4%, 3.1%, 3.7% and 3.1%, respectively.

**Table 1:** The results yielded by SGCLDGA in 5-fold CV

No.	AUC	AUPR	Recall	Precision	F1-score
1	0.8883	0.9086	0.8292	0.8375	0.8281
2	0.8840	0.9083	0.8274	0.8367	0.8261
3	0.8837	0.9071	0.8249	0.8347	0.8236
4	0.8863	0.9079	0.8271	0.8355	0.8261
5	0.8896	0.9110	0.8310	0.8398	0.8299
Avg.	0.8864	0.9086	0.8279	0.8368	0.8268

**Table 2:** The results of 5-fold cross-validation

Models	AUC	AUPR	Recall	Precision	F1-score
LRGCPND	0.7905	0.7941	0.7059	0.7374	0.6959
LightGCN	0.8087	0.8336	0.7574	0.7851	0.7513
LAGCN	0.8273	0.8476	0.7737	0.7784	0.7722
MF	0.8113	0.8514	0.7528	0.7754	0.7476
AGAEMD	0.8546	0.8536	0.8020	0.8067	0.8013
MNGACDA	0.8612	0.8613	0.7791	0.7794	0.7790
SGCLDGA	**0.8864**	**0.9086**	**0.8279**	**0.8368**	**0.8268**

To further demonstrate the predictive capability of SGCLDGA, we divided the dataset into training, testing and validation sets using an 8:1:1 ratio. The model’s performance was evaluated utilizing metrics such as AUC, AUPR, Recall, Precision and F1-score, with the experimental outcomes detailed in Table 3. Additionally, we compared the AUC and AUPR curves of various models, as illustrated in Figure 2C and Figure 2F. The results demonstrate that SGCLDGA achieved the highest scores of 0.8860, 0.9072, 0.8292, 0.8375 and 0.8281 on the AUC, AUPR, Recall, Precision and F1-score metrics, respectively. These scores signify SGCLDGA’s superior performance in predicting gene–drug associations compared with the other five models. The consistent and notable superiority of SGCLDGA across these metrics underscores its efficacy, accuracy and superiority over the existing methodologies.

**Table 3:** Comparison of SGCLDGA with five other models on the validation set

Models	AUC	AUPR	Recall	Precision	F1-score
LRGCPND	0.7904	0.7920	0.6977	0.7343	0.6854
LightGCN	0.8128	0.8417	0.7608	0.7885	0.7549
LAGCN	0.8388	0.0.8547	0.7769	0.7776	0.7768
MF	0.8105	0.8504	0.7518	0.7739	0.7467
AGAEMD	0.8527	0.8467	0.7996	0.8072	0.7984
MNGACDA	0.8598	0.8640	0.7737	0.7731	0.7734
SGCLDGA	**0.8860**	**0.9072**	**0.8292**	**0.8375**	**0.8281**

### Ablation study

To assess the efficacy of the contrastive learning strategy, we perform ablation experiments, and the results are outlined in Table 4. We employ several ablation strategies for experimentation and subsequent comparison.

- **GCN-NONE**: We removed the contrastive learning module and conducted experiments using the traditional GCN method.
- **GCN-ED**: GCL-ED represents edge perturbation, indicating that the graph augmentation method involves perturbing edges.
- **GCN-ND**: GCL-ND stands for node dropping, which means the graph augmentation method is to remove nodes.
- **GCN-RW**: GCL-RW refers to random walk, which implies that the graph augmentation method is based on random walks.

**Table 4:** The results of ablation study

Methods	AUC	AUPR	Recall	Precision	F1-score
GCN-NONE	0.8087	0.8336	0.7574	0.785	0.7513
GCN-ED	0.8398	0.8638	0.7714	0.7941	0.7670
GCN-ND	0.8380	0.8622	0.7622	0.7953	0.7533
GCN-RW	0.8377	0.8619	0.7616	0.7966	0.7544
SGCLDGA	**0.8863**	**0.9076**	**0.8279**	**0.8368**	**0.8268**

As shown in Table 4, contrastive learning methods have certain advantages over traditional graph-based methods. This is because contrastive learning can make the learned representations more evenly distributed in the representation space, thus improving the model’s generalization ability and robustness. The contrastive learning strategy can effectively enhance the performance of SGCLDGA. Compared with the method without contrastive learning, SGCLDGA achieves higher scores of 9.91%, 8.40%, 9.22%, 6.36% and 9.98% on the AUC, AUPR, Recall, Precision and F1-score metrics, respectively. Moreover, our approach surpasses traditional graph perturbation-based contrastive learning methods. This indicates that using SVD to reconstruct the gene–drug association graph and injecting global collaborative context signals is important for learning unified feature representations of genes and drugs, thereby improving the predictive performance of the model.

### Performance and efficiency analysis

Combining the experimental results from Table 2 and Table 4, we draw several conclusions:

- Compared with traditional graph-based (LightGCN,LAGCN) methods, recent contrastive learning-based approaches (GCN-ED, GCN-ND, GCN-RW) demonstrate certain advantages while also outperforming classical collaborative filtering algorithms (MF). This can be attributed to the fact that contrastive learning forces the model to learn more representative feature representations by capturing subtle differences between samples, rather than focusing solely on common features in the data.
- Our method outperforms other state-of-the-art approaches in terms of performance, including other contrastive learning baselines(GCN-ED, GCN-ND, GCN-RW). We credit the performance enhancement to effectively augmenting graph contrastive learning by incorporating global collaborative contextual signals. In contrast, other contrastive learning-based methods in comparison are prone to bias from noisy interaction information, resulting in the generation of misleading self-supervised signals.

We conducted a statistical analysis of the time and memory consumption required for performing 5-fold cross-validation on the models used in the experiments. The experiments were conducted under fair conditions using the same server, without interference from other programs, and with the same epoch set to 300. The results are shown in Table 5.

- The runtime of SGCLDGA is significantly lower compared with methods using similarity measures such as LAGCN, AGAEMD and MNGACDA. This is because when the association matrix becomes larger, methods using similarity measures require a considerable amount of time to compute similarity, leading to longer runtimes.
- GCL models typically incur higher computational costs due to building additional views and performing convolution operations on them during training. However, the low-rank nature of SVD reconstructed graphs and the simplified CL structure make our SGCLDGA training highly efficient. Although our model requires SVD computation in the preprocessing stage, the computational cost compared with the training phase can be negligible as it needs to be executed only once. Indeed, by moving the construction of contrastive views to the preprocessing stage, we avoided repetitive graph enhancement during training, thus improving the efficiency of the model. It can be seen that SGCLDGA saves nearly twice the time compared with other GCL models such as GCN-ED, GCN-ND and GCN-RW.
- Our model’s runtime is almost identical to that of LightGCN, which once again demonstrates that the computational cost of SVD computation during the preprocessing stage can be negligible. Moreover, it highlights that our model’s training efficiency is on par with LightGCN, which is highly scalable and computationally efficient.

**Table 5:** The time and memory cost of the models

**Models**	**Time expenditure**	**Memory cost**
LRGCPND	48 min	131 MB
LightGCN	**16 min**	98 MB
LAGCN	467 min	131 MB
MF	25 min	**65 MB**
AGAEMD	194 min	**65 MB**
MNGACDA	306 min	131 MB
GCN-ED	41 min	131 MB
GCN-ND	48 min	131 MB
GCN-RW	39 min	131 MB
SGCLDGA	**16 min**	131 MB

### Parameter sensitivity analysis

We conducted a parameter sensitivity analysis on SGCLDGA to investigate the impact of the embedding dimension and the number of GNN layers on its predictive performance. While keeping other parameters constant, we systematically varied the embedding dimension and the number of GNN layers, followed by a comprehensive 5-fold cross-validation.

#### Influence of embedding size

**Figure 3:**
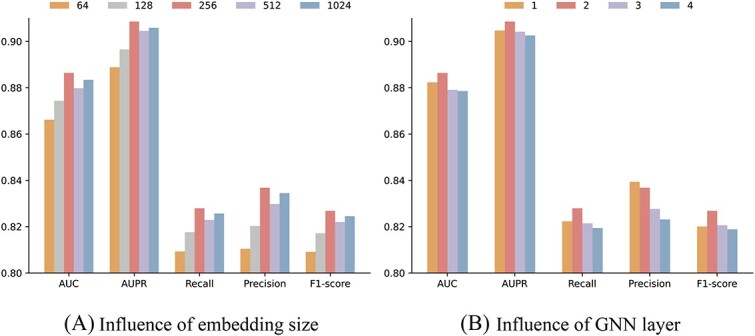
Parameter sensitivity visualization.

We held constant all other parameters while varying the embedding dimension across values of 64, 128, 256, 512 and 1024. Through 5-fold cross-validation, we measured and visualized the AUC, AUPR, Recall, Precision and F1-score, presenting the results in bar charts as illustrated in Figure 3A. Notably, we observed an improvement in SGCLDGA’s performance with an increase in the embedding dimension, reaching its optimum at 256.

#### Influence of GNN layer

We varied the number of GNN layers across values of 1, 2, 3 and 4. Through 5-fold cross-validation, we assessed and visualized the results in bar charts as depicted in Figure 3B. Notably, SGCLDGA demonstrated its optimal performance when the number of GNN layers was set to 2. Further increases in the number of layers resulted in smoother learned features, leading to a loss of crucial information and a subsequent decline in SGCLDGA’s performance.

### Embedding visualization

**Figure 4:**
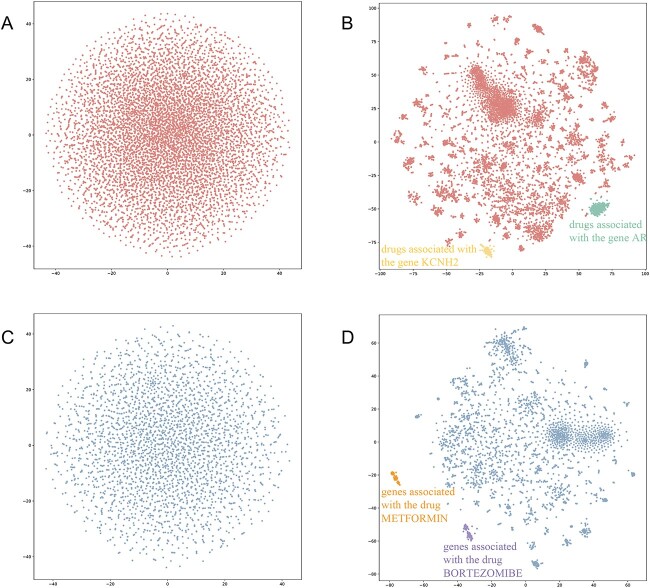
Embedding visualization by t-SNE. The color blue represents drug embedding and the color red represents gene embedding. (**A**) Initial embedding of drug. (**B**) Embedding of drug after being trained by SGCLDGA. (**C**) Initial embedding of gene. (**D**) Embedding of gene after being trained by SGCLDGA.

To assess the impact of SGCLDGA on the distribution of embeddings for drugs and genes, we utilized t-SNE (t-Distributed Stochastic Neighbor Embedding) for visual representation. t-SNE is a technique for reducing the dimensionality of data and creating visualizations in a lower dimensional space, typically two or three dimensions. Figures 4A and 4C depict the distributions of drug and gene embeddings before training, where blue corresponds to drugs and red to genes. Prior to training, the embeddings were observed to be randomly scattered in the 2D space. After applying the SGCLDGA model, the embeddings of drugs and genes, displayed in Figures 4B and 4D, reveal effective clustering of similar drugs and genes. This clustering enhances the identification of potential associations, demonstrating the model’s capacity to organize and group related entities.

First, we select drugs and genes clustered together in the graph and assess whether they have experimentally verified associations with the same drug or gene. As an illustration, we pick two aggregation groups from the visualized graphs of drugs and genes obtained after training the model to assess the effectiveness of the clustering. In Figure 4B, the yellow group contains 127 drugs, and 123 of them are associated with the gene KCNH2. Similarly, the green group comprises 233 drugs, all of which are related to the gene AR. In Figure 4D, the orange group consists of 49 genes, and all of them are associated with the drug METFORMIN. Furthermore, the purple group encompasses 58 genes, and 56 of them are associated with the drug BORTEZOMIBE. This analysis confirms that SGCLDGA is an effective model for identifying similar drugs and genes, as it accurately groups entities that share experimental associations.

### Anti-cancer drug–gene association analysis

**Figure 5:**
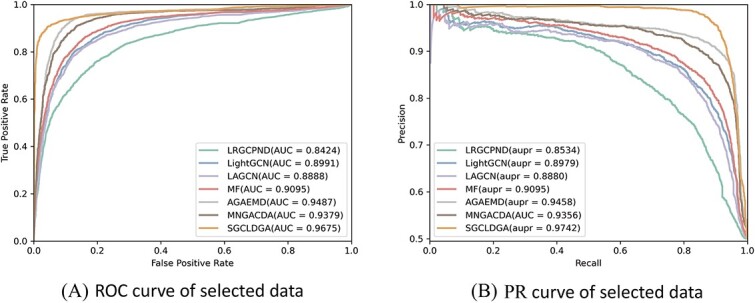
The performance of models in anti-cancer drug–gene association analysis.

Cancer presents a significant global health challenge, driven by genetic mutations that result in abnormal cell growth. Targeted therapies, which manipulate gene expression, highlight the necessity of understanding gene–drug associations to deepen our comprehension of therapeutic mechanisms. Despite SGCLDGA’s promising performance on the DGIdb dataset, we aim to scrutinize its efficacy at a more detailed level to enhance its practical relevance. Anticancer drugs are pivotal in combating cancer, a multifaceted disease that can manifest in various forms across different body parts. These drugs disrupt tumor growth and metastasis by impeding cancer cell proliferation, division and spread through diverse mechanisms. To this end, we meticulously selected 82 anticancer drugs, including AFATINIB and BLEOMYCIN, for evaluation, comparing their predictive performance with previous experiments. Our analysis, presented in Table 6 along with ROC and PR curve plots (Figure 5), unequivocally demonstrates SGCLDGA’s superior performance, showcasing significant enhancements across multiple evaluation metrics. This underscores SGCLDGA’s exceptional ability to predict unknown anticancer drug–gene associations, thereby bolstering its utility in real-world applications.

**Table 6:** The results of anti-cancer drug–gene association analysis

Models	AUC	AUPR	Recall	Precision	F1-score
LRGCPND	0.8423	0.8533	0.7787	0.7787	0.7787
LightGCN	0.8991	0.8978	0.8286	0.8325	0.8281
LAGCN	0.8888	0.8880	0.8227	0.8243	0.8224
MF	0.9095	0.9095	0.8404	0.8445	0.8399
AGAEMD	0.9486	0.9457	0.9026	0.9055	0.9025
MNGACDA	0.9378	0.9356	0.8864	0.8873	0.8863
SGCLDGA	**0.9674**	**0.9741**	**0.9310**	**0.9320**	**0.9309**

### Case study

Exploring the unknown associations between drugs and genes carries significant practical implications. In our endeavor to delve deeper into this realm, we conducted a case study focusing on specific drugs and genes. To further validate the predictive prowess of SGCLDGA in uncovering unknown anti-cancer drug–gene associations, we meticulously selected drugs and genes from distinct disease categories, such as lung cancer and breast cancer. Additionally, we deliberately included a pair of genes and drugs that were exceedingly rare in the dataset for comprehensive study. For the selected drugs, we systematically excluded known associated genes and ranked the remaining genes in descending order based on predictions generated by SGCLDGA. Similarly, for the selected genes, we omitted known associated drugs and ranked the remaining drugs in a similar manner. Subsequently, we identified the top 15 genes and drugs, respectively, and conducted an exhaustive review of the published literature to corroborate our findings with supporting evidence.

Based on disease categories, we focused our study on lung cancer and breast cancer, filtering out pertinent drugs and genes from the dataset. For lung cancer, we selected the drug Afatinib and the gene BRAF as our subjects of interest. Afatinib emerges as a pivotal first-line treatment option for patients afflicted with lung adenocarcinoma, particularly those harboring complex EGFR mutations [45]. Notably, it demonstrates efficacy, especially among individuals exhibiting uncommon mutation patterns. BRAF, on the other hand, represents an oncogene pivotal in cell signaling and regulatory mechanisms. Within the context of lung cancer, BRAF gene mutations have been identified, particularly among patients diagnosed with NSCLC [46]. This underscores its relevance in understanding the molecular landscape and therapeutic interventions in lung cancer cases. For breast cancer, our focus turned to the drug Brivanib and the gene FGFR1. Brivanib stands out as a multi-targeted anti-tumor agent primarily targeting vascular endothelial growth factor receptor (VEGFR) and fibroblast growth factor receptor (FGFR), thereby impeding angiogenesis and tumor cell proliferation [47]. Its potential in breast cancer treatment is noteworthy, given its ability to inhibit crucial pathways implicated in tumor growth. FGFR1, encoding Fibroblast Growth Factor Receptor 1, emerges as a pivotal gene in breast cancer pathogenesis. Aberrations in FGFR1 expression or mutations thereof are intricately linked to the onset and progression of breast cancer, underscoring its significance as a therapeutic target and prognostic marker [48]. Moreover, we deliberately selected a set of drugs and genes that are seldom encountered in the dataset: ACRIDINE and A2M. These selections serve to broaden the scope of our study, allowing us to explore potential associations and shed light on less-explored facets of drug–gene interactions in cancer biology.

Tables 7, 8 and 9 present case studies focusing on drugs and genes associated with lung cancer, breast cancer and less studied subjects, respectively. Notably, the prediction accuracy for unknown drug–gene associations concerning AFATINIB, BRAF, BRIVANIB, FGFR1, ACRIDINE and A2M reached impressive percentages of 86.7%, 80%, 73.3%, 80%, 66.7% and 86.7%, respectively. These results underscore the robust predictive capability of SGCLDGA in identifying unknown drug–gene associations within the context of cancer treatment. Furthermore, SGCLDGA demonstrates commendable predictive performance even when confronted with less frequently occurring drugs and genes in the dataset. However, it is essential to acknowledge that while these findings are promising, there may exist additional associations yet to be confirmed. This emphasizes the imperative for further experimental investigations to elucidate and validate these potential relationships, thereby enhancing our understanding of cancer biology and treatment mechanisms.

**Table 7:** Predicted associations in lung cancer: genes associated with AFATINIB and drugs associated with BRAF

**Gene**	**PMID**	**Drug**	**PMID**
PTEN	26934000	VANDETANIB	36624452
PIK3CA	30630630	CARBOPLATIN	30383888
MAP2K1	35898964	GEMCITABINE	28500236
ALK	30568455	PACLITAXEL	30383888
RAF1	34648945	DOCETAXEL	24567366
MAP2K2	35004247	APITOLISIB	28779636
FGFR2	35420673	OLAPARIB	Unconfirmed
FGFR1	25115383	CRIZOTINIB	34516041
KDR	26681011	SIROLIMUS	Unconfirmed
HRAS	38237027	TOZASERTIB	35197630
SRC	32064003	SUNITINIB	25841455
CDKN2A	Unconfirmed	AZD-8055	24879157
STK11	29575851	WX-554	Unconfirmed
YES1	32744377	HESPERADIN	37770979
RET	Unconfirmed	TAE-684	21847362

**Table 8:** Predicted associations in breast cancer: genes associated with BRIVANIB and drugs associated with FGFR1

**Gene**	**PMID**	**Drug**	**PMID**
PDGFRB	28728751	LINIFANIB	29367740
PDGFRA	28728751	IMATINIB	25673643
KIT	24710173	ERLOTINIB	30639621
FLT3	31186890	GEMCITABINE	38387284
CSF1R	31508222	DACTOLISIB	36276073
RET	28626729	EVEROLIMUS	26503204
SRC	35600776	SITRAVATINIB	37469409
LCK	Unconfirmed	CARBOPLATIN	31446228
LYN	22005529	CETUXIMAB	26416732
ABL1	Unconfirmed	BOSUTINIB	Unconfirmed
AXL	31684985	CHEMBL406845	Unconfirmed
PTK2	23991421	PACLITAXEL	38137377
AURKB	Unconfirmed	DORAMAPIMOD	37879219
VEGFA	24710173	AZD-1152-HQPA	Unconfirmed
YES1	Unconfirmed	QUIZARTINIB	32021432

**Table 9:** Predicted associations of less studied drug and gene: genes associated with ACRIDINE and drugs associated with A2M.

**Gene**	**PMID**	**Drug**	**PMID**
ACE	32151835	CISPLATIN	22141344
NOS1	Unconfirmed	HEPARIN	2482548
SLC2A4	Unconfirmed	PROGESTERONE	21978460
BDNF	16957779	GEMCITABINE	35281857
AGT	Unconfirmed	ASPIRIN	30943276
MMP2	Unconfirmed	TAMOXIFEN	26657294
BAX	16177561	STAUROSPORINE	31115360
ADRB2	36376373	FLUOROURACIL	6578156
ADRB1	37890443	DOXORUBICIN	25818003
CSF2	Unconfirmed	ANDROSTANOLONE	32085797
IL2	2528623	DEXAMETHASONE	2435908
DDIT3	32836316	INSULIN	2470761
MYC	12034846	MELATONIN	37108290
GFAP	17662460	HYDROCORTISONE	Unconfirmed
BCL2	11042675	REGRAMOSTIM	Unconfirmed

## DISCUSSION AND CONCLUSION

Traditional drug development and discovery have long been recognized as laborious, resource-intensive and fraught with risks. In contrast, drug repurposing offers a compelling strategy to circumvent these challenges. By unveiling drug–gene interactions, we not only unearth new therapeutic uses for existing drugs but also identify potential targets for novel treatments. However, the conventional approach to biological experimentation is marked by its time-consuming nature, exorbitant costs and susceptibility to environmental influences. Fortunately, the advent of multi-source information has paved the way for the development of efficient and cost-effective computational methods. While GCN-based models have demonstrated considerable promise in predicting drug–gene associations, they predominantly rely on supervised learning paradigms. This reliance renders them particularly vulnerable to the issue of data sparsity, a common hindrance in real-world drug discovery endeavors. In practice, sparse data often translate into suboptimal outcomes, thereby underscoring the pressing need for innovative solutions to this challenge. To address these issues, we propose SGCLDGA, a novel contrastive learning-based method designed for predicting potential drug–gene associations. In the SGCLDGA framework, random initializations of gene and drug embeddings are obtained from the dataset. Leveraging gene–drug associations, a bipartite gene–drug graph is constructed, and GCN is employed to iteratively update the embeddings of gene and drug nodes. Additionally, SGCLDGA performs graph reconstruction using SVD and introduces a contrastive learning paradigm that optimizes the node embeddings by contrasting the original and reconstructed graphs. The final step involves employing inner product operations to derive association scores for specific gene–drug pairs. Experimental results showcase the superior performance of SGCLDGA compared with state-of-the-art methods, with ablation studies confirming the individual contributions of each component in SGCLDGA. Case studies further demonstrate the efficacy of SGCLDGA in predicting potential gene–drug associations.

Our future directions involve incorporating more independent data sources and integrating additional biological contextual knowledge. Additionally, we aim to explore the implementation of transfer learning techniques to enhance the model’s generalization ability and further mitigate the impact of data sparsity on predictive performance.

**Key Points**

- We have developed a lightweight but effective framework for predicting drug–gene associations, addressing key challenges in the field.
- Our model, SGCLDGA, guided by singular value decomposition, efficiently preserves vital gene–drug association information, avoiding the need for manually crafted views.
- Compared with existing methods, SGCLDGA significantly improves training efficiency and outperforms similarity-based approaches, especially with larger datasets.
- Extensive experiments validate the superior performance of SGCLDGA, confirming its rationale and practical value.

## Author Biographies

**Yanhao Fan.** is a graduate student at School of Computer, Central South University, Changsha, China. His research interests include machine learning and bioinformatics.

**Che Zhang** is a graduate student at School of Computer, Xinjiang University, Urumqi, China. Her research interests include machine learning and data mining.

**Xiaowen Hu** is a graduate student at School of Computer, Central South University, Changsha, China. His research interests include machine learning and bioinformatics.

**Zhijian Huang** is a graduate student at School of Computer, Central South University, Changsha, China. His research interests include machine learning and bioinformatics.

**Jiameng Xue** is a undergraduate student at School of Computer, Central South University, Changsha, China. His research interests include machine learning and bioinformatics.

**Lei Deng** is a professor at School of Computer Science and Engineering, Central South University, Changsha, China. His research interests include data mining, bioinformatics and systems biology.
